# Complete mitochondrial genome sequence of yellowtail catfish *Pangasius pangasius*

**DOI:** 10.1128/mra.01366-25

**Published:** 2026-05-28

**Authors:** Md. Mahmud Hasan, Moonmoon Ahamed Mim, Md. Sagir Ahmed, Sabuj Biswas

**Affiliations:** 1 Fisheries Biotechnology Division, National Institute of Biotechnology 563134, Savar, Dhaka, Bangladesh; 2 Advanced Fisheries and DNA Barcoding Laboratory, Department of Zoology, University of Dhaka 95324, Dhaka, Bangladesh; University of California Riverside, Riverside, California, USA

**Keywords:** mitochondrial genome, yellowtail catfish, *Pangasius pangasius*

## Abstract

We report the first complete mitochondrial genome sequence of *Pangasius pangasius* from Bangladesh. The 16,472 bp circular genome contains 13 protein-coding genes, 22 tRNA genes, two rRNA genes, and one control region, and its guanine-cytosine (GC) content is 44.4%.

## ANNOUNCEMENT

*Pangasius pangasius*, commonly known as yellowtail catfish, is a species belonging to the Pangasiidae family distributed in the rivers of South and Southeast Asia (1). Despite its economic and nutritional importance, overexploitation and habitat loss have made this species critically endangered in Bangladesh (2). Prior research has delineated the mitochondrial genomes of *P. pangasius* populations originating from India (3). However, there is a lack of information regarding the mitochondrial DNA of *P. pangasius* from Bangladesh. To address this gap, this paper presents the full mitochondrial genome sequence of *P. pangasius* from Bangladesh, offering significant genetic insights for molecular phylogeny, evolutionary and biogeographic linkages, aquaculture management, and conservation research.

A dead specimen of *P. pangasius* was collected and sourced from the Andharmanik River (21.854°N, 90.124°E) on 10 August 2023. Mitochondrial DNA (mtDNA) was extracted from the tissue of the pectoral fin using the mtDNA isolation kit (Abcam, ab65321, USA). Quality and quantity of mtDNA were measured by a NanoDrop 2000 (Thermo Fisher, USA) and visualized in 1% agarose gel. Next-generation sequencing (NGS) libraries were prepared using the Nextera XT DNA library preparation kit, and sequencing was performed by Illumina NovaSeq X Plus from Macrogen, Korea, yielding 151 bp long, paired-end reads. A total of 32,230,432 raw reads were generated, which corresponded to 4.9 Gbp of sequencing data, and the depth of coverage was 100×. FastQC version 0.12.1 (4) was used to evaluate the quality of raw reads, and TrimGalore version 0.6.10 (5) was used to filter out low-quality data (Q < 20). The genome was assembled using NOVOPlasty v4.3.1 (6), where *Pangasius pangasius* (KX950698.1) was used as the seed sequence. A 16,472 bp single mt-genome contig was obtained, which was coordinated and circularized by MitoAnnotator (7). The genome was annotated using MITOS2 (8), and overlaps were checked manually. Finally, the circular map was generated and visualized using circularMT (9).

**Fig 1:**
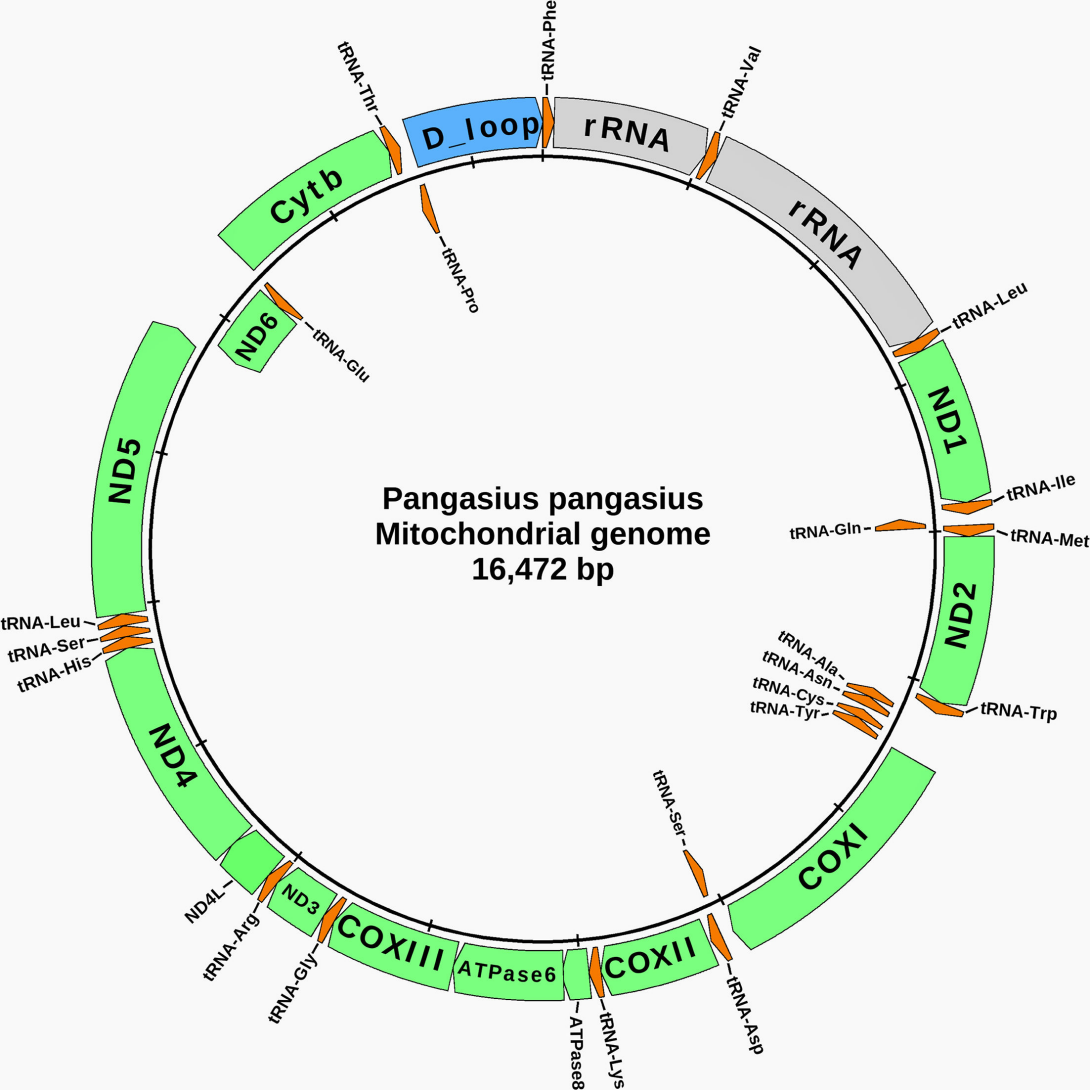
Circular mitochondrial genome map of *P. pangasius*. The annotated map comprises 13 protein-coding genes, 22 transfer RNA genes, two ribosomal RNA genes (12S and 16S), and the putative control region (D-loop). Genes transcribed clockwise are on the outer circle, indicating the heavy strand, and counterclockwise genes are on the inner circle, indicating the light strand. Protein-coding genes, rRNAs, and tRNAs are shown in green, gray, and orange, respectively. The complete map was generated and visualized using circularMT.

The 16,472 bp complete mitogenome of *P. pangasius* has one non-coding control region (D-loop), two rRNA (12S and 16S rRNAs), 22 tRNAs, and 13 protein-coding genes (PCGs) (Fig. 1). The majority of genes are encoded on the heavy (H) strand, whereas the light (L) strand contains the nad6 and eight tRNA genes (Table 1). The start codons for all PCGs are ATG, except COX-I, which initiates with GTG. Termination codons include TAA, TAG, or incomplete stop codons (T- or TA-). The rRNA genes are 957 bp (*rrnS*) and 1,659 bp (*rrnL*) in length. The control region is located between *trnP* and *trnF*. The base composition of the mitochondrial genome is as follows: A = 5,021 (30.5%), T = 4,140 (25.1%), C = 4,726 (28.7%), and G = 2,585 (15.7%), with a total of 44.4% guanine-cytosine (GC) content. Compared with two previously deposited *P. pangasius* mitochondrial genomes of Indian sources from National Center for Biotechnology Information (NCBI), this assembly showed 99.88 and 99.47% identity to the accession numbers KX950698.1 and NC_023924.1, respectively. The newly sequenced mitogenome from Bangladesh provides significant genomic insights, which will facilitate future studies on the evolutionary history, species identification, and population genetics of *P. pangasius* and its related species.

**TABLE 1:** Mitochondrial genome content, organization, and codon information of *P. pangasius*

Gene	Location	Gene length (bp)	Start codon	Stop codon	Anti-codon	H/L strand^*b*^
trnF	1–70	70			GAA	+
12S rRNA	71–1027	957				+
trnV	1028–1099	72			TAC	+
16S rRNA	1123–2781	1659				+
trnL2	2780–2854	75			TAA	+
nad1	2855–3829	975	ATG	TAA		+
trnL	3831–3902	72			GAT	+
trnQ	3902–3972	71			TTG	−
trnM	3972–4041	70			CAT	+
nad2	4042–5088	1047	ATG	T^*a*^		+
trnW	5087–5157	71			TCA	+
trnA	5160–5228	69			TGC	−
trnN	5230–5302	73			GTT	−
trnC	5334–5400	67			GCA	−
trnY	5403–5472	70			GTA	−
cox1	5474–7024	1551	GTG	TAA		+
trnS2	7025–7095	71			TGA	−
trnD	7100–7172	73			GTC	+
cox2	7187–7877	691	ATG	T^*a*^		+
trnK	7878–7951	74			TTT	+
atp8	7953–8120	168	ATG	TAA		+
atp6	8111–8794	684	ATG	TA^*a*^		+
cox3	8794–9578	785	ATG	T^*a*^		+
trnG	9578–9650	73			TCC	+
nad3	9651–10001	351	ATG	T^*a*^		+
trnR	10000–10070	71			TCG	+
nad4L	10071–10367	297	ATG	TAA		+
nad4	10361–11741	1381	ATG	T^*a*^		+
trnH	11742–11811	70			GTG	+
trnS1	11812–11878	67			GCT	+
trnL1	11881–11953	73			TAG	+
nad5	11954–13780	1827	ATG	TAA		+
nad6	13777–14295	519	ATG	TAG		−
trnE	14296–14364	69			TTC	−
cob/cytb	14366–15503	1138	ATG	T^*a*^		+
trnT	15504–15575	72			TGT	+
trnP	15574–15643	70			TGG	−
D-loop	15644–16472	829				+

^*a*^ Truncated termination codon.

^*b*^ +, heavy (H) strand; −, light (L) strand.

## Data Availability

The complete mitochondrial genome sequence of *P. pangasius* has been deposited in the GenBank database under accession number PV699345. The version described in this manuscript is the first version, PV699345.1. The raw sequencing data of this study have been deposited in the NCBI Sequence Read Archive (SRA) under accession number SRR35764323 (BioProject accession number PRJNA1339031 and BioSample accession number SAMN52624715).
